# Breast carcinomas with osteoclast-like giant cells: a comprehensive clinico-pathological and molecular portrait and evidence of RANK-L expression

**DOI:** 10.1038/s41379-022-01112-9

**Published:** 2022-06-13

**Authors:** Joanna Cyrta, Camille Benoist, Julien Masliah-Planchon, Andre F. Vieira, Gaëlle Pierron, Laetitia Fuhrmann, Camille Richardot, Martial Caly, Renaud Leclere, Odette Mariani, Elisabeth Da Maia, Frédérique Larousserie, Jean Guillaume Féron, Matthieu Carton, Victor Renault, François-Clément Bidard, Anne Vincent-Salomon

**Affiliations:** 1 Department of Pathology, Institut Curie, PSL Research University, Paris, France; 2 Université de Paris, Paris, France; 3 Clinical Bioinformatics Unit, Institut Curie, PSL Research University, Paris, France; 4 Somatic Genetics Unit, Institut Curie, Paris, France; 5 Platform of Experimental Pathology PATHEX, Institut Curie, Paris, France; 6 Department of Pathology, Hôpital de la Pitié-Salpêtrière, Paris, France; 7 Department of Pathology, Hôpital Cochin, AP-HP, Université Paris Cité, Paris, France; 8 Department of Surgery, Institut Curie, Paris, France; 9 Department of Biometry, DRCI, Institut Curie, PSL Research University, Paris, France; 10 Department of Medical Oncology, Institut Curie, UVSQ/Paris-Saclay University, St Cloud, France

**Keywords:** Breast cancer, Osteoclasts, Breast cancer, Cancer genetics, Tumour biomarkers

## Abstract

Breast carcinomas (BC) with osteoclast-like giant cells (OGC) are rare. Despite their distinct stromal features, their molecular characteristics remain unknown. Here, we report comprehensive clinico-pathological and molecular findings for 27 patients diagnosed with BC-OGC at Institut Curie between 2000 and 2021. Seventeen (63%) cases were invasive carcinomas of no special type (IC NST) with OGC (OGC-IC NST), four (15%) were mixed or multifocal cases with and without OGC (OGC-Mixed), and six (22%) were metaplastic carcinomas with OGC (OGC-MC). All OGC-IC NST and OGC-Mixed cases were ER+ HER2− tumors (most being luminal A based on transcriptomic subtyping, when available), while all OGC-MC were triple-negative. The median age at diagnosis was 46, 45 and 62 years for OGC-IC NST, OGC-Mixed and OGC-MC, respectively. Three patients developed distant metastases (one OGC-IC NST, two OGC-Mixed), one of whom died of metastatic disease (OGC-Mixed), and one other patient died of locally advanced disease (OGC-MC). Histopathological evaluation comparing 13 OGC-IC NST and 19 control IC NST without OGC confirmed that OGC-IC NST showed significantly higher density of vessels (by CD34 immunohistochemistry (IHC)), iron deposits (Perls stain), and CD68 and CD163-positive cell infiltrates. Genomic findings for nine OGC-IC NST and four OGC-MC were consistent with the underlying histologic subtype, including activating alterations of the PI3K/AKT/mTOR pathway in 7/13 cases. Using RNA-seq data, differential gene expression analysis between OGC-IC NST (*n* = 7) and control IC NST without OGC (*n* = 7) revealed significant overexpression of *TNFSF11* (RANK-L), *TNFRSF11A* (RANK), *CSF1* (M-CSF), *CSF1R*, and genes encoding osteoclastic enzymes (*MMP9, ACP5*, *CTSK*, *CTSB*) in OGC-IC NST, while *OPG* (osteoprotegerin) was underexpressed. We also confirmed for the first time RANK-L expression in BC with OGC by IHC (seen in 15 out of 16 cases, and only in 2 of 16 controls without OGC). These findings could offer a rationale for further investigating RANK-L as a therapeutic target in BC with OGC.

## Introduction

Breast carcinomas (BC) with osteoclast-like giant cells (OGC) are a rare entity. They are characterized by the presence of OGC within the tumor and currently considered as a special morphologic pattern rather than a stand-alone entity by the 2019 WHO breast tumor classification^1^. Although the presence of OGC has most often been reported in invasive carcinomas of no special type (IC NST), it can also occur in other subtypes of BC^1–4^. In addition to OGC, recurrent stromal features have been described in these tumors, including inflammatory infiltrate, hypervascular stroma, and erythrocyte extravasation^1,2,4,5^. Despite these distinctive features, the molecular underpinnings of this morphologic pattern have not been elucidated to date. In addition, due to the rarity of these tumors, comprehensive studies of larger cohorts are lacking.

The goal of this study was to report a detailed clinicopathologic description of BC with OGC diagnosed at our institution, a formal analysis of their stromal features, as well as their genomic and transcriptomic characterization, with the aim of identifying potential molecular events that could explain the presence of OGC.

## Materials and methods

### Patients and samples

We performed a retrospective search of the electronic patient database (ConSoRe) of Institut Curie (Paris, France), using the keywords “breast”, “carcinoma” and “osteoclast(ic)” to identify patients whose medical records contained all three terms. Those patients who had undergone breast surgery between 2000 and 2021 and for whom pathology slides were available at Institut Curie were included. Slides were reviewed by study pathologists (J.C., A.V.S.) to confirm the diagnosis. Clinical data were obtained from electronic medical records and/or from treating oncologists. The study was approved by the Institutional Review Board (reference: DATA220058).

### Pathology data

All cases were further classified into three groups: (1) invasive carcinomas of no special type with OGC (group 1: OGC-IC NST); (2) “mixed” cases, i.e., tumors composed of clearly distinct areas with and without OGC, or multifocal tumors with and without OGC (group 2: OGC-Mixed); and (3) metaplastic carcinomas (MC) with OGC (group 3: OGC-MC). Diagnoses of IC NST and MC were made in accordance with criteria of the 2019 WHO breast tumor classification^1^. Surgical pathology data (size, grade, lymph node status, hormone receptors, HER2 and Ki67 status) were collected from pathology reports and/or during pathology review.

### Histopathologic evaluation of stromal features

Vascular density in tumors was assessed using CD34 immunohistochemistry (IHC). Density of hemosiderin/iron deposits was evaluated using Perls Prussian blue stain. The overall density of monocyte/macrophage infiltrates was evaluated using CD68 and CD163 IHC, and expression of these two markers was also assessed specifically in OGC.

IHC was performed using the Dako Autostainer Link 48 (Agilent Technologies, Santa Clara, CA) with the following antibodies and conditions: anti-CD34 (clone QBEnd 10 [Dako, Stockholm, Sweden], 1/100 dilution, pH9 20 min retrieval); anti-CD68 (clone KP1 [Dako], 1/800 dilution, pH9 20 min retrieval); anti-CD163 (clone 10D6 [Novocastra, Leica], 1/100 dilution, pH6 20 min retrieval). All slides were scanned on a Hamamatsu Nanozoomer S360 scanner (Hamamatsu, Japan), ×40 objective, and staining quantification was performed using Qupath (v0.3.2) software^6^. The invasive tumor area, including a 1 mm rim of peri-tumor tissue, was manually selected. Areas devoid of tissue (artefacts, glandular lumina, adipocytes) were automatically excluded using a pixel classifier, and areas of coarse artefacts or necrosis were manually excluded. For each staining, the optimal positivity threshold was set in such a way as to minimize false positive signal, and the same threshold was used across all cases. Results for each stain were expressed as a percentage of the positive area within the area of interest, and comparison between subgroups was made using the Wilcoxon signed-rank test and considering *p* < 0.05 as significant. CD68 and CD163 expression specifically in OGC was evaluated by a pathologist (J.C.) in a semi-quantitative manner.

### Whole exome sequencing

Whole exome sequencing (WES) was performed on DNA extracted from fresh-frozen tumor tissue and on matched germline DNA. Libraries were prepared using the SureSelect Agilent XT2 kit (Agilent Technologies, Santa Clara, CA) and sequencing was performed on an Illumina NovaSeq 6000 platform (Illumina, San Diego, CA) in paired-end 100 base-pair (bp) mode. Read alignment was performed using bwa (v.0.7.15) on the hg19 human assembly. Reads cleaning was done as described by GATK best practices recommendations (v3.5). Variant calling was performed using Haplotype Caller and Mutect2 (GATK3.5) and variant annotation with ANNOVAR (v2018Apr16). Copy number alterations (CNA) were analyzed using Sequenza (v.2.1.0).

### Targeted panel-based DNA sequencing

DNA extracted from formalin-fixed paraffin-embedded (FFPE) tumor tissues was sequenced using a custom next generation sequencing panel of 571 genes. Briefly, indexed paired-end libraries were prepared using ~100 ng of DNA with the Agilent SureSelect XT kit (Agilent Technologies) and sequenced on a NovaSeq 6000 Sp2x100 bp flow cell (Illumina). The bioinformatics pipeline has been described previously^7^.

### RNA sequencing

Total RNA were extracted from fresh-frozen tumor tissues using the Qiagen RNAeasy kit (Qiagen, Venlo, the Netherlands). Libraries were prepared using the Illumina TruSeq Stranded mRNA Library preparation kit and sequencing was performed on an Illumina NovaSeq 6000 platform in paired-end 100 bp mode. Fusion transcript detection was done using Defuse (0.6.0), FusionMap (2015-01-09), FusionCatcher (0.99.7d) and STAR-Fusion (1.0.0). Expression data were generated with SALMON (v 0.13.1) on ensembl96 (hg19) and count matrices were normalized as Transcripts Per Million using tximport (R library).

Molecular BC subtyping was performed using the subtype.cluster.predict() function (sbt.model = “pam50”) from R package *genefu* (version 2.16.0), based on the intrinsic subtyping classifier that measures expression of 50 genes (PAM50) selected as characteristic of the five BC intrinsic subtypes^8^.

Principal component analysis was performed on the top 5000 genes with the highest variance. Differential expression analysis was done with the R DESeq2 package, considering adjusted *p* value <0.05 as significant.

Gene set enrichment analysis (GSEA) was performed with the GSEA application for Mac (v4.2.3), using a pre-ranked list of a total of 14,630 genes. Gene Ontology overrepresentation analysis was performed using the enrichGO() function with ont = “BP” (for biological processes), “MF” (for molecular functions), “CC” (for cellular components) from R package *clusterProfiler*.

Additional details of the statistical, genomic and transcriptomic analyses are provided in Supplementary Methods.

### Immunohistochemistry for RANK-L

IHC for RANK-L was performed on sections of FFPE tumor tissues with the M366 mouse monoclonal antibody^9,10^ (Amgen, Thousand Oaks, CA), using a Bond RX automated immunostainer (Leica Microsystems, Buffalo Grove, IL) and the Bond Polymer Refine detection system (Leica). Heat-mediated antigen retrieval was performed with Bond ER2 solution at pH9 for 20 min. The M366 antibody was used at a concentration of 4.4 μg/ml, with 1 h of incubation. The IHC protocol was validated using giant cell tumors of the bone as positive controls and a microarray of benign tissues as negative controls (Supplementary Fig. S1). Staining was assessed in invasive carcinoma and, when available, in the associated in situ component and in benign epithelial structures on the same slide. The percentage of positive cells, staining intensity and subcellular staining location were evaluated by a pathologist (J.C.) blinded to the histologic subgroup of each case. Results between subgroups were compared using the Wilcoxon signed-rank test, considering *p* < 0.05 as significant.

## Results

### Clinicopathologic data

Twenty-seven patients were identified between 2000 and 2021; this represents ~0.05% of patients who underwent surgery for invasive BC at our institution during that period. Seventeen (63%) cases were classified as IC NST with OGC (OGC-IC NST), four (15%) as “mixed” cases (OGC-Mixed), and six (22%) as metaplastic carcinomas with OGC (OGC-MC). A summary of clinicopathologic findings for each group is presented in Table 1. Additional details are provided in Supplementary Tables ST1 and ST2.

**Table 1:** A summary of clinico-pathological data for the cohort.

Category	OGC-IC NST (*n* = 17)	OGC-Mixed (*n* = 4)^a^	OGC-MC (*n* = 6)
Age at diagnosis: median (range) (years)	46 (33–68)	45 (44–48)	62 (38–84)
Personal history of breast cancer	3 (18%)	–	2 (33%)
pT stage			
pT1b	2 (12%)	–	–
pT1c	9 (53%)	–	1 (17%)
pT2	6 (35%)	4 (100%)	5 (83%)
Lymph node status			
N0	14 (82%)	2 (50%)	4 (67%)
N0(i+)	–	–	1 (17%)
N+	3 (18%)	2 (50%)^c^	1 (17%)
Elston-Ellis grade			
I	9 (53%)	1 (25%)	–
II	7 (41%)	3 (75%)	–
III	1 (6%)	–	5 (83%)
Not available	–	–	1 (17%)
Phenotype (IHC)			
ER+	17 (100%)	4 (100%)	–
PR+	17 (100%)	4 (100%)	–
HER2+	–	–	–
Triple-negative	–	–	6 (100%)
Ki67 index: median (range)	10% (1–30%)	11% (10–25%)	55% (40–70%)
Follow up: median (range) (months)	79 (5–151)	21 (13–161)	38.5 (6–230)
Neoadjuvant chemotherapy	1 (6%)	–	–
Surgery			
Lumpectomy	15 (88%)	2 (50%)	3 (50%)
Total mastectomy	2 (12%)	2 (50%)	3 (50%)
Adjuvant treatment(s)			
Chemotherapy	5 (29%)	2 (50%)	5 (83%)
Radiation therapy	16 (94%)	3 (75%)	4 (67%)
Hormone therapy	15 (88%)	3 (75%)	–
Recurrence during follow-up			
No recurrence	15 (88%)	2 (50%)	4 (67%)
Local recurrence	2 (12%)	–	2 (33%)^d^
Distant metastases	1 (6%)^b^	2 (50%)	–
Clinical status at the end of follow-up			
Alive disease-free	16 (94%)	2 (50%)	5 (83%)
Alive with metastases	1 (6%)	1 (25%)	–
Died of disease	–	1 (25%)	1 (17%)

*IC NST* invasive carcinoma of no special type, *MC* metaplastic carcinoma, *ILC* invasive lobular carcinoma, *DCIS* ductal carcinoma in situ.

^a^Unifocal mixed IC NST with OGC and ILC without OGC (*n* = 1); multifocal IC NST with and without OGC (*n* = 2); multifocal ILC without OGC and IC NST with OGC (*n* = 1).

^b^Same patient who earlier developed local recurrence.

^c^Includes one micrometastasis.

^d^In one case, the local recurrence was DCIS only.

**Fig. 1:**
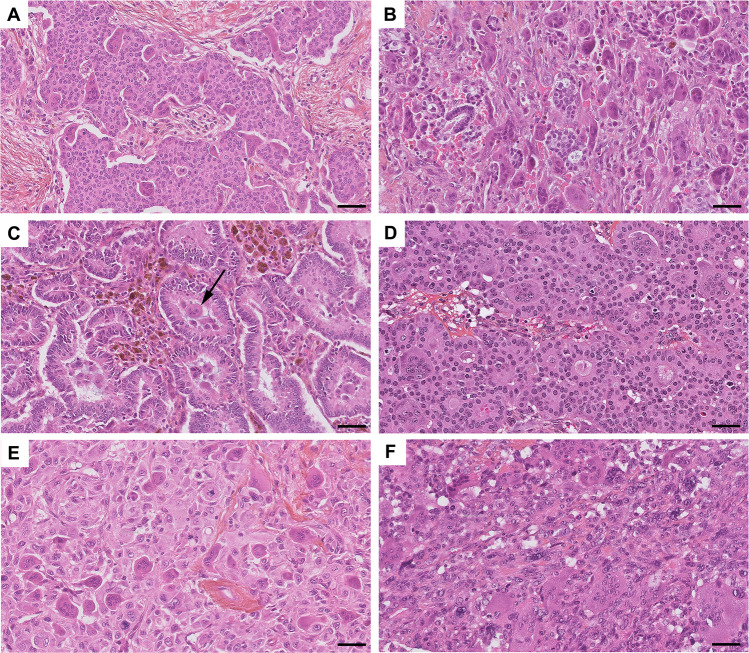
Examples of histomorphologic patterns in BC with OGC. **A** Solid pattern (case OGC-IC NST 4); **B** discohesive tumor clusters, associated with numerous OGC and stromal hemorrhage (case OGC-IC NST 5); **C** tubular and “colonic-like” architecture (case OGC-IC NST 1), with OGC in tubular lumens (arrow); **D** cribriform architecture with OGC embedded in glandular lumens (case OGC-IC NST 12); **E** metaplastic carcinoma with OGC (OGC-MC 2) showing spindle cell and squamous features; **F** metaplastic carcinoma with OGC (OGC-MC 3) showing spindle cell features. Hematoxylin-eosin-saffron stain. Scale bars: 50 μm.

A variety of morphologies was observed (Fig. 1). OGC-IC NST displayed tubular, cribriform, trabecular and/or solid architecture; two cases had a minor micropapillary component. Within the OGC-Mixed group, there were two cases of multifocal IC NST with and without OGC, one multifocal case including IC NST with OGC and multiple foci of invasive lobular carcinoma (ILC) without OGC, and one case of mixed NST and ILC with OGC present only in a part of the IC NST component.

In a subset of cases, OGC could readily be seen in the associated in situ component (Supplementary Fig. S2A). OGC were also present, at least focally, in the four lymph node metastases that could be reviewed (Supplementary Fig. S2B, C).

Gross pathology description and/or photographs were available for eight OGC-IC NST, seven of which (87.5%) showed a brownish, hemorrhagic, or “rusty” appearance (Supplementary Fig. S3 and Supplementary Table ST2).

By IHC, all OGC-IC NST and OGC-Mixed were ER+, HER2− tumors, and most (*n* = 13 and *n* = 2, respectively) had a Ki67 index <20%. Conversely, all OGC-MC were triple-negative tumors with elevated Ki67 indices (range: 40–70%).

### Histopathological evaluation of stromal features

**Fig. 2:**
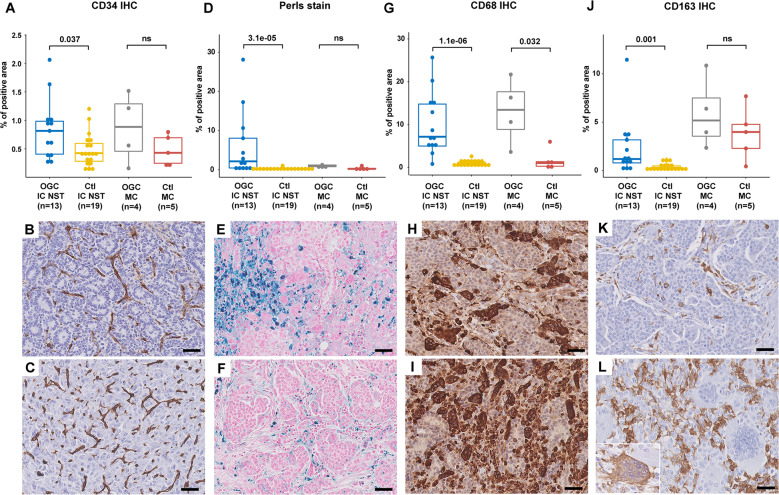
Histopathologic evaluation of stromal features in BC with OGC. **A** Quantification of anti-CD34 IHC; examples of CD34 staining in OGC-IC NST 1 (**B**) and in OGC-MC 2 (**C**), showing high vascular density; **D** quantification of Perls staining; examples of Perls stain in OGC-IC NST 5 (**E**) and OGC-IC NST 4 (**F**); **G** quantification of anti-CD68 IHC**;** examples of CD68 IHC in OGC-IC NST 6 (**H**) and in OGC-IC NST 5 (**I**); of note, all OGC were positive; **J** quantification of anti-CD163 IHC; examples of CD163 IHC in OGC-IC NST 4 (**K**) and in OGC-MC 1 (**L**); most OGC were negative, with rare exceptions (inset). Scale bars: 50 μm. Boxplots: median, interquartile range; Wilcoxon test.

Immunostains for CD34, CD68 and CD163, as well as Perls stain, were quantified across 13 cases of OGC-IC NST, 4 cases of OGC-MC, 19 control IC NST without OGC (Ctl-IC NST) (selected to show IHC subtypes, grade and stage comparable to those of OGC-IC NST), and 5 control MC without OGC (Ctl-MC). Results are presented in Fig. 2 and in Supplementary Table ST3. Mixed cases were not included in this evaluation.

The percentage of tumor area positive for CD34, considered as a surrogate for vascular density, was significantly higher in OGC-IC NST than in Ctl-IC NST (mean: 0.8% vs. 0.5%, *p* = 0.037) (Fig. 2A). A similar tendency was observed for OGC-MC vs. Ctl-MC, but this comparison was not statistically significant. The percentage of area positive with Perls stain, considered as a surrogate for past stromal hemorrhage events, was also significantly higher in OGC-IC NST than in Ctl-IC NST (mean, 5.9% vs. 0.3%, *p* < 0.001) (Fig. 2D), although positivity of this stain was not a consistent feature in all BC with OGC. Erythrocyte extravasation (Supplementary Fig. S4 and Supplementary Table ST3) was seen in 14 of the 16 (87.5%) OGC-IC NST, and in only 2 of the 19 Ctl-IC NST (10.5%) for which it could be assessed (Fisher’s exact test: *p* < 0.0001).

We next evaluated the density of CD68+ and CD163+ cell infiltrates. As expected, the proportion of tumor area positive for CD68 was significantly higher in OGC-IC NST than in Ctl-IC NST (mean: 10.1% vs. 0.9%, *p* < 0.001), and in OGC-MC compared to Ctl-MC (mean: 13.1% vs. 1.7%, *p* = 0.032) (Fig. 2G). The percentage of area positive for CD163 was also significantly higher in OGC-IC NST than in Ctl-IC NST (mean: 2.3% vs. 0.4%, *p* = 0.001), while the comparison was not statistically significant for MC (Fig. 2J).

OGC consistently displayed strong and diffuse CD68 expression in all OGC-IC NST and OGC-MC (Fig. 2H, I). Conversely, CD163 positivity was only observed in rare individual OGC (estimated to represent 1% or less of the OGC population) in 2 OGC-MC and in 2 OGC-IC NST (Fig. 2K, L and Supplementary Table ST3).

### Genomic findings

WES was performed on 7 OGC-IC NST and on one OGC-MC, as well as on matched germline DNA. Targeted panel-based DNA sequencing was performed on additional two OGC-IC NST and three OGC-MC. The most salient findings are summarized in Table 2. Additional results are available in Supplementary Tables ST4 and ST5 and in Supplementary Figs. S5 and S6.

**Table 2:** A summary of the most salient genomic findings in BC with OGC.

Case	EE	Test	TC	Alterations (mutations or CNA)				Tumor VAF	COSMIC occurrence in breast cancers^b^
OGC-IC TNS 1	1	WES	36%	1p loss, 1q gain/16q loss, 5q loss, 6q loss, 11p gain, 11q loss, chr20 gain				–	–
OGC-IC TNS 2	2	WES	46%	*PIK3CA*	Missense	c.3140A > G	p.(His1047Arg)	32%	2228
				3p loss				–	–
OGC-IC TNS 3	3	WES	64%	*PIK3CA*	Missense	c.1624G > A	p.(Glu542Lys)	48%	624
				*MAP2K4*	Stop gain	c.979C > T	p.(Gln327*)	69%	1
				Multiple CNA, including 3N/4N ploidy				–	–
OGC-IC TNS 4	2	WES	59%	3p loss, 7q loss				–	–
OGC-IC TNS 5	1	WES	41%	1q gain/16q loss, 6q loss, 22q loss				–	–
OGC-IC TNS 6	1	WES	25%	*MAP3K1*	Stop gain	c.3595C > T	p.(Gln1199*)	18%	3
				“Simplex” profile, no evident CNA				–	–
OGC-IC TNS 7	2	WES	12%	1q gain, 13q gain, gain of chromosomes 11, 14, 16, 19, 21				–	–
OGC-IC TNS 12	1	Panel	62%	*GNAS*	Missense	c.601C > T	p.(Arg201Cys)	29%	8
				16p gain, 16q loss, 14q loss (partial), chr 21 gain				–	–
OGC-IC TNS 13	1	Panel	62%	1q gain/16q loss, 2q loss, 5p gain, 5q loss (partial), 16p gain, 19p gain				–	–
OGC-MC 1	3	WES	20%	*AKT1*	Missense	c.49G > A	p.(Glu17Lys)	10%	374
				*BRAF*	Missense	c.1799T > A	p.(Val600Glu)	19%	0
				8q gain				–	–
OGC-MC 2	3	Panel	45%	*PIK3CA*	Missense	c.1258T > C	p.(Cys420Arg)	50%	51
				*TP53*	Frameshift	c.636del	p.(Arg213Asp_fs)	42%	1
				1p gain, 3q gain^a^				–	–
OGC-MC 3	3	Panel	59%	*HRAS*	Missense	c.37G > C	p.(Gly13Arg)	48%	2
				*PIK3R1*	inframe_del	c.1700_1711del	p.(Lys567_Leu570del)	16%	2
				*TP53*	Splice site	c.782 + 1G > T	p.?	10%	6
				7q loss, 8p loss, 8q gain, 12q loss, 17p loss, 17q loss (partial)				–	–
OGC-MC 6	3	Panel	28%	*PIK3CA*	Missense	c.3140A > G	p.(His1047Arg)	14%	1556
				*PTEN*	Missense	c.395G > A	p.(Gly132Asp)	25%	1
				6p gain, 15p loss, 10q copy-neutral LOH				–	–

*EE* Elston-Ellis grade, *TC* tumor cellularity (as estimated by bioinformatic pipelines), *CNA* copy number alterations, *VAF* variant allele frequency, *LOH* loss of heterozygosity.

^a^“Noisy” CNA profile, only the most obvious alterations are noted.

^b^As of March 2022.

In terms of CNA, most OGC-IC NST (*n* = 7, 78%) showed diploid, “simplex” genomes. Concomitant 1q gain/16q loss, a recurrent alteration previously reported for grade 1 luminal A tumors^11^, was seen in three cases (OGC-IC NST 1, 5 and 13). Two cases (OGC-IC NST 3 and 7) showed more complex profiles. In OGC-IC NST 3 (a luminal B, grade 3 IC NST), most of the genome was tri- or tetraploid. In OGC-MC, we observed some CNA previously described as recurrent in MC^12,13^, including 8q gain (*n* = 2), 3q gain (*n* = 1), 8p loss (*n* = 1), 7p loss (*n* = 1), and 12q loss (*n* = 1).

No high-level amplifications in known oncogenes were evidenced in the cohort.

Among all sequenced BC with OGC (*n* = 13), several single nucleotide variations (SNV) in genes known to be recurrently altered in BC were identified. Hotspot *PIK3CA* mutations were seen in four cases (30%, two OGC-IC NST and two OGC-MC). We also observed truncating mutations in *MAP3K1* and *MAP2K4* (each in one OGC-IC NST), a hotspot activating *AKT1* mutation (one OGC-MC), and an in-frame deletion in *PIK3R1* (one OGC-MC). Deleterious *TP53* alterations (frameshift and splice site) were seen in one OGC-MC each, but not in OGC-IC NST. Other alterations in known cancer genes included a *BRAF* hotspot V600E mutation in one OGC-MC. *GNAS* and *HRAS* hotspot mutations (one OGC-IC NST and one OGC-MC, respectively), and a probably pathogenic *PTEN* variant (one OGC-MC). No recurrent SNV which could be pathognomonic for BC with OGC were identified.

### Transcriptomic findings

RNA-seq was performed on fresh-frozen tissue from seven OGC-IC NST, two OGC-MC, seven control IC NST cases without OGC (Ctl-IC NST) (selected to show IHC subtypes, grade and stage comparable to those of OGC-IC NST), and two control MC cases without OGC (Ctl-MC).

We did not identify recurrent fusion transcripts that could be pathognomonic of BC with OGC with the methods used herein (data not shown).

Based on molecular subtype classification (PAM50 genes), six of the seven OGC-IC NST (86%) were luminal A tumors, one (OGC-IC NST 3) was a luminal B tumor, and the two OGC-MC were basal-like tumors.

**Fig. 3:**
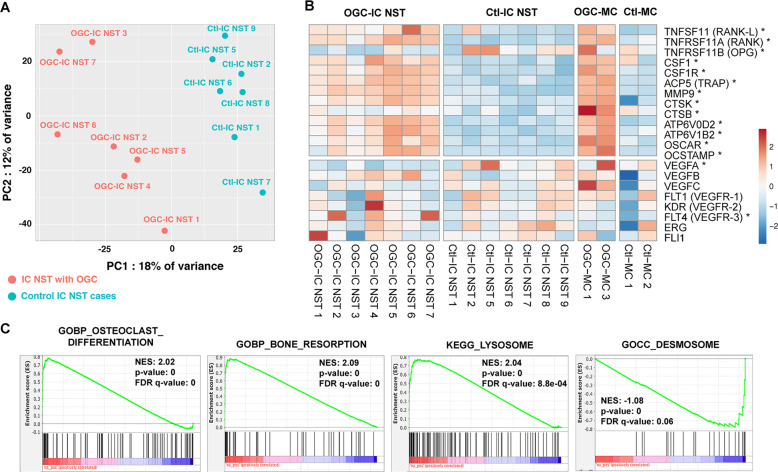
Selected transcriptomic findings in BC with OGC. **A** Principal component analysis using the top 5000 genes with the highest variance. **B** Heatmap representation of log2 normalized TPM values of 21 selected genes for 18 samples; rows (genes) are centered and scaled to show the differences between the conditions; *indicates genes that were significantly differentially expressed (adjusted *p* < 0.05) between OGC-IC NST and Ctl-IC NST; the top 13 genes are related to osteoclast differentiation or function, the bottom eight are related to angiogenesis. **C** Gene set enrichment analysis (GSEA) plots for selected gene sets.

When looking at gene expression levels of OGC-IC NST and Ctl-IC NST, Principal Component Analysis revealed a separation between the two groups (Fig. 3A) with the first two components, suggesting differences in gene expression profiles. Differential gene expression analysis was next performed between these two groups (Supplementary Tables ST6 and ST7), and 1402 significantly differentially expressed genes (adjusted *p* value <0.05) were identified. Most notably, OGC-IC NST showed significant overexpression of genes involved in osteoclast differentiation as compared to Ctl-IC NST, including *TNFSF11* (which encodes RANK-L), *TNFSFR11A* (RANK), *CSF1* (M-CSF) and *CSF1R* (Fig. 3B). Conversely, *OPG* (encoding osteoprotegerin, a soluble decoy receptor which neutralizes RANK-L and inhibits RANK-L/RANK signaling) was significantly underexpressed in OGC-IC NST. Other genes significantly overexpressed in OGC-IC NST included genes encoding osteoclastic enzymes, such as *MMP9* (matrix metallopeptidase 9), *ACP5* (tartrate-resistant protein phosphatase, or TRAP), *CTSK* and *CTSB* (Cathepsin K and B, respectively), and genes encoding V-ATPases. Of note, a similar expression pattern for these genes (except for *OPG*) was observed in OGC-MC (Fig. 3B), but the sample size was insufficient for differential expression analysis.

GSEA and Gene Ontology overrepresentation showed significant enrichment of gene sets relative to macrophage differentiation, osteoclast maturation, bone resorption/remodeling, phagocytosis, lysosome function, and pH reduction in OGC-IC NST (Fig. 3C, Supplementary Figs. S7–S9, and Supplementary Tables ST8 and ST9). Gene sets with negative enrichment scores included those relative to desmosomes, cytoskeleton components, and epithelial maturation.

When querying individual genes involved in angiogenesis, we observed significantly lower *VEGFA* gene expression in OGC-IC NST than in the control group, significantly higher *VEGFR3* expression in OGC-IC NST, and no statistically significant difference for *VEGFB, VEGFC, VEGFR1* or *VEGFR2* genes. GSEA and Gene Ontology analyses did not show significant enrichment of gene sets relative to angiogenesis in OGC-IC NST.

### RANK-L protein expression in BC with OGC

**Fig. 4:**
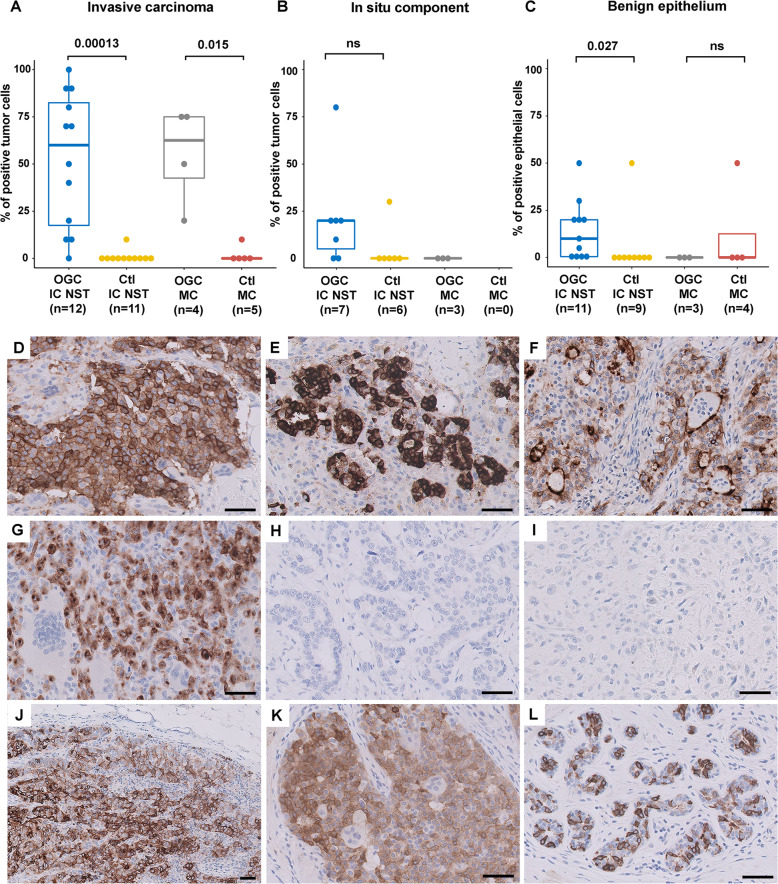
Evaluation of RANK-L IHC in BC. Summary of RANK-L IHC results (Wilcoxon test) across subgroups for invasive carcinoma (**A**), associated DCIS component (**B**) and associated benign epithelium (**C**); **D** OGC-IC NST 6 showing diffuse, moderate to strong membranous and cytoplasmic RANK-L expression; note that the OGC are negative; **E** OGC-IC NST 5 showing diffuse and particularly strong membranous and cytoplasmic expression; **F** OGC-IC NST 12 showing apical membrane reinforcement of RANK-L expression; note that the OGC are negative and preferentially located in the vicinity of the apical membranes of tumor cells; **G** OGC-MC 1 showing cytoplasmic RANK-L expression and para-nuclear dots; note that the OGC are negative; Examples of control cases; Ctl-IC NST 3 (**H**) and Ctl-MC 3 (**I**), both completely negative for RANK-L expression; **J** axillary lymph node metastasis in patient OGC-CI NST 5, showing RANK-L expression in metastatic tumor cells; **K** associated DCIS component in OGC-IC NST 6, showing positive RANK-L expression in tumor cells; note that the OGC are negative; **L** associated benign structures in OGC-IC NST 6, showing heterogeneous positivity for RANK-L in benign epithelial cells. OGC IC NST: invasive ductal carcinoma of no special type with OGC; Ctl IC NST: invasive ductal carcinoma of no special type without with OGC; OG-MC: metaplastic carcinoma with OGC; Ctl-MC: metaplastic carcinoma without with OGC; bars, 50 μm. Boxplots: median, interquartile range; Wilcoxon test.

Results of RANK-L IHC evaluation are presented in Fig. 4, Supplementary Tables ST10 and ST11, and Supplementary Figs. S10 and S11.

Remarkably, of the 12 OGC-IC NST and 4 OGC-MC tested, positivity of invasive carcinoma cells for RANK-L was observed in all but one case, with a mean of 52.5% of positive tumor cells (range: 0–100%). All positive cases showed moderate to strong staining intensity. The observed staining patterns in tumor cells included: cytoplasmic staining, para-nuclear dots, circumferential membranous staining, apical membrane staining (1 case), apical vesicles (1 case), or a combination of the above (Fig. 4D–G). Of note, the OGC themselves were consistently negative for RANK-L.

Conversely, among 11 control IC NST and 5 control MC cases, only one IC NST and one MC showed focal positivity for RANK-L (in about 10% of tumor cells in each case). The difference in terms of percentage of positive tumor cells between the OGC-IC NST and Ctl-IC NST groups was highly significant (mean: 52.5% vs. 0.9%, respectively; *p* < 0.001). A statistically significant difference was also noted between MC-OGC and Ctl-MC, despite the small sample size (mean: 55% vs. 2%, respectively; *p* = 0.015).

RANK-L protein expression was also seen in the three lymph node metastases of IC NST with OGC that were tested (range, 40–70% of positive tumor cells) (Fig. 4J).

In addition, in five out of seven OGC-IC NST cases harboring a ductal carcinoma in situ (DCIS) component, we observed RANK-L positivity in the associated DCIS (Fig. 4B, K), but only in one DCIS out of six IC TNS without OGC; statistical tests for this comparison could not be performed due to the small sample size.

Positivity in benign epithelial structures for RANK-L was seen in 8 out of 11 OGC-IC NST cases (Fig. 4C, L), but in only one of 9 IC TNS without OGC (mean, 14.2% vs. 5.6% of positive epithelial cells, *p* = 0.027).

Lastly, among the three OGC-Mixed cases that were tested, some degree of RANK-L positivity was also seen in the component/tumor focus without OGC (range: 5–50%, mean: 20%). However, in those cases, the percentage of positive tumor cells appeared higher and the staining intensity stronger in the component with OGC than in the component without OGC (Supplementary Fig. S11).

## Discussion

### Clinico-pathological characteristics of BC with OGC

Based on our study, and in line with the literature^1,5^, BC with OGC are a rare entity. Nevertheless, their true incidence may be somewhat underestimated, because retrospective identification of cases is hampered by the lack of a specific disease code, and because the presence of OGC could occasionally be missed or underreported on histologic examination^14^.

The most frequent histologic type associated with OGC in our cohort was IC NST (63% of the total cohort, and 78% if “mixed” cases are included). This is consistent with the literature: among 166 BC with OGC reported across 51 publications (Supplementary Table ST12), 110 (66.3%) were IC NST. Nevertheless, OGC have also been described in special types of BC (about 11.4% of published cases), including ILC, cribriform carcinoma, and other types^1–4^. In addition, six cases (22%) from our cohort were MC with OGC. Association of OGC with MC has been previously reported, with at least 37 cases documented in the literature (~22% of published cases of BC with OGC), although most (*n* = 29) as part of a single cohort^15^. It is particularly important that pathologists be aware of this entity, as it could represent a diagnostic pitfall and should not be misdiagnosed as primary breast sarcoma or giant cell tumor of the soft tissue.

Our IHC and gene expression data confirm that OGC-IC NST are ER+ HER2− tumors, and that most belong to the luminal A subtype. This is in keeping with previous IHC findings^5,16^, and with microarray expression profiling of 5 OGC-IC NST from one study^17^. Conversely, all OGC-MC from our cohort were triple-negative tumors, as expected for this subtype.

Our data suggest that OGC-IC NST are diagnosed at a relatively young age (median, 46 years), in line with previous publications^2,4,5,14,18,19^. It is therefore all the more striking that these tumors consistently display a luminal phenotype, as one would expect to also encounter triple-negative and HER2-enriched tumors in patients aged <50 years^20^. The choice of tamoxifen as initial hormone therapy in 17 of 18 patients (90%) (Supplementary Table ST1) suggests that they were pre-menopausal at diagnosis. Altogether, it is possible that OGC-IC NST could represent a particular type of luminal tumors, perhaps occurring in a specific clinical and hormonal setting. Conversely, OGC-MC were diagnosed in older patients (median, 62 years), in line with the average age of diagnosis reported for MC^21,22^.

Fifteen (88.2%) of the 17 patients with “pure” OGC-IC NST from our cohort showed no evidence of disease for a median follow-up of 75 months. Nevertheless, two patients (11.8%) experienced local recurrence, one of whom (5.9%) developed distant metastases 11 years from initial diagnosis. This patient had a grade 3, luminal B tumor with a relatively complex genomic profile. Similarly, Zhou et al. reported lung metastases in two of 35 patients (5.7%) with OGC-IC NST at 7 and 11 years of follow-up^5^. In addition, in the “mixed” group in our study, one patient died of metastatic disease 12 years from initial diagnosis (OGC-Mixed 1), and one developed distant metastases at 6 months of follow-up (OGC-Mixed 4); however, these observations may be confounded by the possible contribution of the component without OGC to metastatic progression. Lastly, in the OGC-MC group, one patient (16.7%) died in a context of locally advanced disease (chest wall extension). Taken together, our findings further support the hypothesis that prognosis of BC with OGC depends on the underlying histo-molecular subtype, rather than on the presence of OGC alone^1^. This could explain why BC with OGC were found to behave aggressively in early series that included both OGC-IC NST and OGC-MC^2^, while more recent studies focusing on OGC-IC NST report favorable outcomes^5^. Nevertheless, the possibility of late metastatic recurrence in rare cases of OGC-IC NST should be kept in mind.

### Genomic findings in BC with OGC

To our knowledge, this study is the first to report next generation sequencing results for BC with OGC. The CNA profiles of BC with OGC from our cohort were consistent with the underlying histologic subtype^11–13^. This is in keeping with a previous study, which found CGH-array based genomic profiles of 4 OGC-IC NST to be consistent with ER+, non-high grade IC NST^23^. We observed frequent activating alterations of the PI3K/AKT/mTOR pathway in BC with OGC (54% of cases overall), which is a known phenomenon in luminal IC NST^24,25^, but has also been reported in MC^12^. The fact that no *TP53* mutations were found in the OGC-IC NST cases is consistent with the predominantly luminal A subtype of these tumors^25^. Some additional alterations were also identified in individual cases, e.g., a *BRAF* hotspot V600E mutation in one OGC-MC, previously reported in ~2–3% of triple-negative BC^26^. Overall, genomic findings in BC with OGC appear consistent with the underlying histologic subtype. No novel genomic alteration or fusion transcript which could be characteristic of BC with OGC could be evidenced in this study.

### The stromal phenotype of BC with OGC

Our study provides the first systematic quantitative evaluation of stromal features in BC with OGC, offering an objective validation of previously described findings. First, using the CD34 immunostain as a surrogate marker, we showed that the proportion of tumor area occupied by vessels was statistically higher in OGC-IC NST and in OGC-MC than in their respective controls without OGC. This is consistent with “hypervascular” stroma classically described in BC with OGC^2,5,14,27^, and with one study which performed a vascular count for two cases^28^. In addition, our study confirms frequent erythrocyte extravasation, and evidence of past hemorrhagic events (iron deposits) in BC with OGC. These microscopic findings are in accordance with the hemorrhagic, brownish or “rusty” gross appearance of these tumors^5,18,29^.

To elucidate the hypervascular nature of BC with OGC, it was hypothesized that tumor cells may secrete pro-angiogenic factors. Expression of VEGF-A by IHC has been reported in four BC with OGC, but these results are difficult to interpret given the small sample size and the absence of experimental controls^28,30,31^. Conversely, our differential gene expression analysis showed significantly lower *VEGFA* gene expression in OGC-IC NST than in the control group. We did observe a significantly higher *VEGFR3* expression in OGC-IC NST, albeit mainly driven by three cases with strong expression. *VEGFR3* encodes a receptor that is not only expressed by endothelial cells, but also by osteoclasts, and it can stimulate bone resorption upon VEGF-C binding^32^. We did not observe significant differences in expression levels of other selected pro-angiogenic factors, or enrichment of angiogenesis pathways. Taken together, additional studies are needed to confirm whether an upregulation of pro-angiogenic factors is indeed a feature of BC with OGC.

CD68 is widely used as a monocyte/macrophage marker, but is not entirely specific of this lineage; conversely, CD163 shows higher specificity^33^, but is also considered to be a marker of alternatively activated, “M2-polarized” macrophages, which have been described as anti-inflammatory or pro-tumoral^34^. In a 2018 study of eight BC with OGC, Ohashi et al. reported most OGC to be CD163+ by IHC^16^. In contrast to their results, we found an overwhelming proportion of OGC to be CD163-negative (Fig. 2), similar to a recent case report^31^, and consistent with the expected phenotype of osteoclasts^35^. However, we did observe a significantly higher density of mononuclear CD163+ infiltrate in BC with OGC than in the control group, and identified individual CD163-positive OGC in four cases. Thus, at least a subset of OGC could potentially derive from CD163+ macrophages, but the CD163+ phenotype does not seem to be maintained in OGC.

### Transcriptomic findings and evidence of RANK-L expression

To our knowledge, this is the first study to report significantly deregulated gene expression programs between OGC-IC NST and IC NST without OGC. One limitation of our analysis is the fact that bulk RNA-seq does not allow one to determine which cell type is responsible for the significantly deregulated transcripts. Nevertheless, a few hypotheses can be proposed.

First, our data suggest that OGC in BC do not merely resemble osteoclasts, but also share their functions, including matrix degradation, as exemplified by overexpression of genes such as *ACP5, MMP9, CTSK*, as well as by GSEA and Gene Ontology results. Accordingly, expression of selected osteoclastic enzymes has previously been evidenced in BC-associated OGC by IHC^16,28,36,37^, and one functional in vitro study confirmed that these cells were capable of bone resorption^37^.

Second, our results support a deregulation of the RANK-L/RANK/OPG and CSF1/CSF1R pathways, which are involved in macrophage activation and osteoclast formation, in BC with OGC. RANK and CSFR1 receptors are expressed at the surface of osteoclastic precursors, while their respective ligands, RANK-L and CSF, can be expressed at the surface of some cell types (osteoblasts, lymphocytes) or secreted^38^. RANK/RANK-L and CSF1/CSF1R interaction promotes proliferation and survival of osteoclastic precursors, as well as their fusion and differentiation into functional osteoclasts^38^. Conversely, OPG is a soluble decoy receptor, which neutralizes RANK-L and inhibits RANK-L/RANK signaling. Interestingly, Lau et al. have previously shown that macrophages isolated from BC tissue are capable of osteoclast differentiation in vitro, but that this process requires the presence of RANK-L and CSF1, and can be inhibited by OPG^39^.

Critically, we were able to demonstrate for the first time that nearly all BC with OGC show RANK-L protein expression in tumor cells, while this was almost never observed in the control cohort. In previous studies of BC (presumably without OGC), RANK-L expression was found to be positive by IHC in a subset of cases (6–16%, depending on the study)^40–42^. Although correlations between RANK-L expression in BC and clinicopathologic factors were inconsistent across these studies, some authors found it to be associated with a younger patient age, a luminal A phenotype, and pregnancy^42,43^. RANK-L expression was previously tested in a single case of BC with OGC and reported to be negative^44^, but the authors did not provide a validation of the IHC assay used in their study.

Of note, the RANK-L/RANK axis is a key player in mammary gland development^45^. In mice, RANK-L is expressed by luminal cells under the influence of progesterone and can act on mammary stem cells in a paracrine fashion to promote their proliferation and resistance to apoptosis^46,47^. The RANK-L/RANK axis has also been implicated in mammary oncogenesis and in epithelial-to-mesenchymal transition, and pharmacological RANK-L inhibition was shown to reduce breast cancer formation in mouse models^47–50^.

While the functional consequences of RANK-L expression in BC with OGC remain to be investigated, our findings could offer novel therapeutic opportunities, because of the existence of clinically approved RANK-L targeting treatments such as denosumab. Based on the above-cited studies, one possibility is that tumor cells could depend on paracrine RANK-L/RANK signaling for their growth or survival. An additional possibility is that the OGC themselves could secrete pro-tumoral factors, similar to what has recently been described in giant cell tumors of the bone^51^.

Given that the majority of BC with OGC show favorable outcomes, they do not require alternative treatment strategies. However, investigating additional therapeutic options may be warranted in rare cases of OGC-IC NST with metastatic recurrence and in MC with OGC. Moreover, carcinomas with OGC are known to occur in other anatomic sites, such as the pancreas (undifferentiated pancreatic carcinoma with OGC), biliary tract, urinary tract or the thyroid, and these entities can be associated with poor prognosis^52,53^. Thus, our results could provide a rationale for investigating RANK-L expression in these tumor types.

### Proposed models to explain the phenotype of BC with OGC

**Fig. 5:**
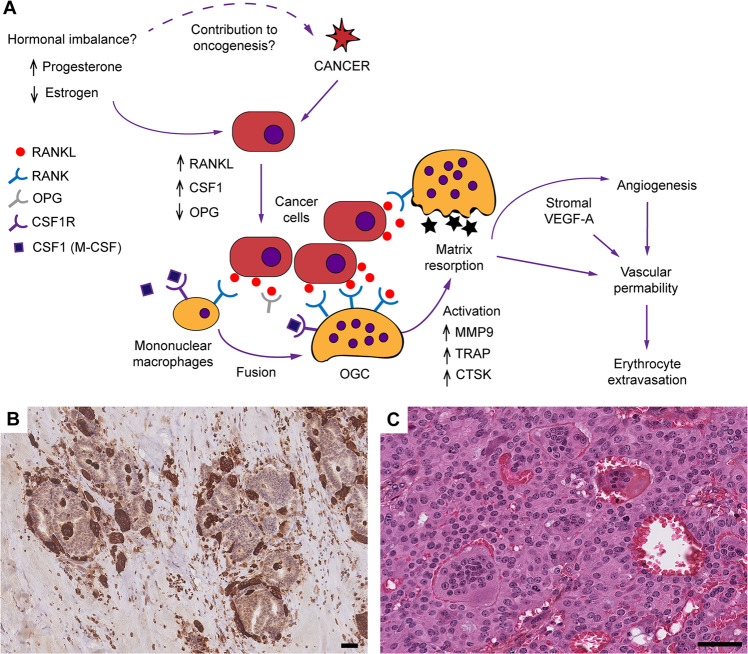
Proposed hypotheses to explain the phenotype of IC NST with OGC. **A** Schematic representation of signaling between tumor cells and macrophages, leading to OGC formation; **B** example of macrophages and OGC showing “molding” around tumor cell clusters (case OGC-IC NST 5; stain, anti-CD68 IHC); **C** example of vascular lacunae sometimes observed in the vicinity of OGC (case OGC-IC NST 12, HES stain). OGC osteoclast-like giant cells. Scale bars, 50 μm. **B**, **C** additional data on these features are provided in Supplementary Table ST3 and Supplementary Fig. S12.

Although these hypotheses remain to be investigated in further studies, our findings suggest some functional models that could help explain the phenotype of BC with OGC (Fig. 5A). First, macrophage fusion and differentiation into OGC could be attributed, at least in part, to an interaction between RANK-L produced by tumor cells and RANK expressed by macrophages. The fact that OGC are always observed in the immediate vicinity of tumor cells (Fig. 5B) might support this hypothesis.

Second, deregulation of the RANK-L/RANK/OPG axis could be related to a particular hormonal state of breast tissue in these patients. For example, RANK-L mRNA expression levels in breast tissue were found to be positively correlated with serum progesterone levels^43,54^. Conversely, OPG expression is stimulated by estradiol and is negatively associated with the progesterone/estradiol balance^38^. Intriguingly, in three cases of BC with OGC, fluctuation in tumor size during menstrual cycles has been reported^4,55^. The fact that we observed RANK-L expression in benign epithelium adjacent to OGC-IC NST significantly more often than in the control cases (73% vs. 11%, Fisher’s test: *p* = 0.009), could further support the hypothesis that OGC-IC NST occur in a specific hormonal setting, which affects not only tumor cells, but a whole “field” of mammary tissue or the entire mammary gland, and promotes RANK-L expression. In OGC-MC, the mechanisms leading to RANK-L expression could be different than in IGC-IC NST (e.g., an aberrant activation of mesenchymal gene expression programs).

Lastly, it was previously noted that OGC are preferentially located in areas of stromal hemorrhage^2,18,19^, and that their number is positively associated with a more vascular/hemorrhagic stroma^14^. One possibility is that macrophages and/or OGC participate in resorption of hemorrhagic debris. However, while siderophages are frequently encountered in these tumors, the OGC themselves contain little or no hemosiderin^55,56^. As such, stromal hemorrhage in BC with OGC could also be a result, rather than the cause, of the presence of OGC. For example, MMP-9-mediated extracellular matrix degradation has been shown to liberate matrix-sequestered VEGF-A, which in turn could increase both angiogenesis and vascular permeability^57^. Hemorrhagic lacunae, which can occasionally be seen around OGC (Fig. 5C), albeit are not a consistent finding, could be in line with this hypothesis.

In conclusion, our study suggests that most BC with OGC are luminal A IC NST occurring in relatively young women, and a subset are triple-negative MC, a potential diagnostic pitfall. It also shows that mutations and CNA in BC with OGC are consistent with the underlying histological subtype. Lastly, it provides evidence for the implication of the RANK-L/RANK/OPG and CSF1/CSF1R pathways in this phenotype. While most BC with OGC carry good prognosis, the rationale for using RANK-L as a potential therapeutic target in rare patients with aggressive disease, as well as in other cancer types with OGC, could warrant further investigation.

## Supplementary information

Supplementary figures

Supplementary tables

Supplementary methods

## Data Availability

Raw RNA-seq and WES data have been deposited into the European Genome-phenome Archive (EGA ID: EGAS00001006238 and EGAS00001006239, respectively). Processed data are available in Supplementary Material.
