# Supplementary material for: Breast carcinomas with osteoclast-like giant cells: a comprehensive clinico-pathological and molecular portrait and evidence of RANK-L expression
Source: Mod Pathol. 2022 Jun 13;35(11):1624–35. doi: 10.1038/s41379-022-01112-9 (PMC9596373; doi:10.1038/s41379-022-01112-9)
Supplement: Supplementary file 1 — Supplementary figures [file 41379_2022_1112_MOESM1_ESM.docx]

**Supplementary figures**

**Breast carcinomas with osteoclast-like giant cells: a comprehensive clinico-pathological and molecular portrait and evidence of RANK-L expression**

Joanna Cyrta^1,2,@^, Camille Benoist^3^, Julien Masliah-Planchon^4^, Andre F. Vieira^1^, Gaëlle Pierron^4^, Laetitia Fuhrmann^1^, Camille Richardot^1^, Martial Caly^1^, Renaud Leclere^1,5^, Odette Mariani^1^, Elisabeth Da Maia^6^, Frédérique Larousserie^7^, Jean Guillaume Féron^8^, Matthieu Carton^9^, Victor Renault^3^, François-Clément Bidard^10^, Anne Vincent-Salomon^1,@^

^1^Department of Pathology, Institut Curie, PSL Research University, Paris, France

^2^Université de Paris, Paris, France

^3^Clinical Bioinformatics Unit, Institut Curie, PSL Research University, Paris, France

^4^Somatic Genetics Unit, Institut Curie, Paris, France

^5^Platform of Experimental Pathology PATHEX, Institut Curie, Paris, France.

^6^Department of Pathology, Hôpital de la Pitié-Salpêtrière, Paris, France

^7^Department of Pathology, Hôpital Cochin, AP-HP, Université Paris Cité, Paris, France

Hôpital Cochin, Paris, France

^8^Department of Surgery, Institut Curie, Paris, France

^9^Department of Biometry, DRCI, Institut Curie, PSL Research University, Paris, France

^10^Department of Medical Oncology, Institut Curie, UVSQ/Paris-Saclay University, St Cloud, France

^@^ Correspondence to: joanna.cyrta@curie.fr and anne.salomon@curie.fr

**Supplementary Figure S1**


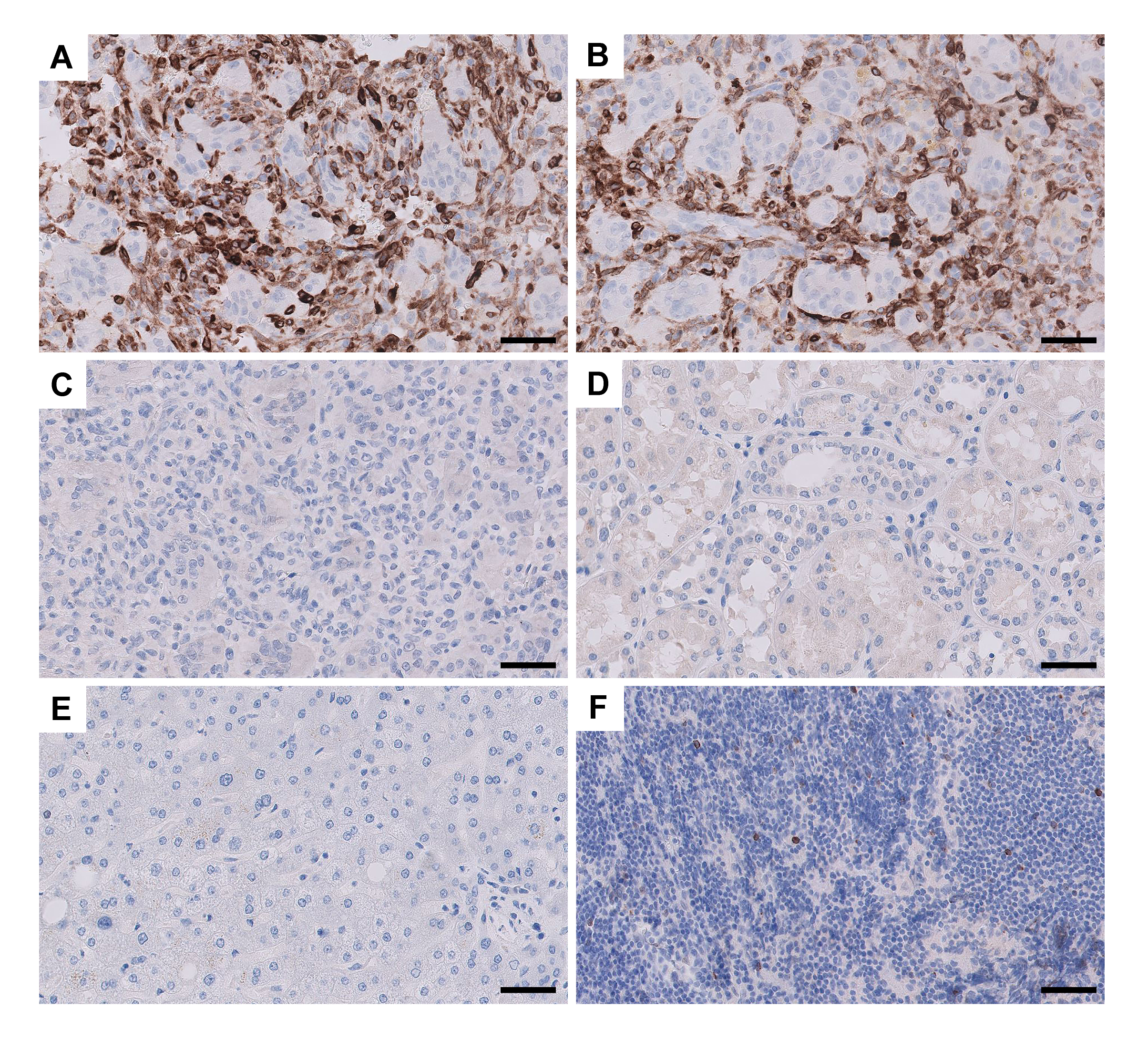


**Supplementary Figure S1. Validation of the IHC protocol for RANK-L using the M366 primary antibody.** **A** and **B:** positive controls - two different cases of giant cell tumor of the bone; **C:** isotype control (mouse IgM applied instead of the primary anti-RANK-L antibody) in a giant cell tumor of the bone; **D-E:** benign tissue controls (**D:** kidney, **E:** liver, **F:** lymphoid tissue). Scale bars, 50 μm.

**Supplementary Figure S2**

**
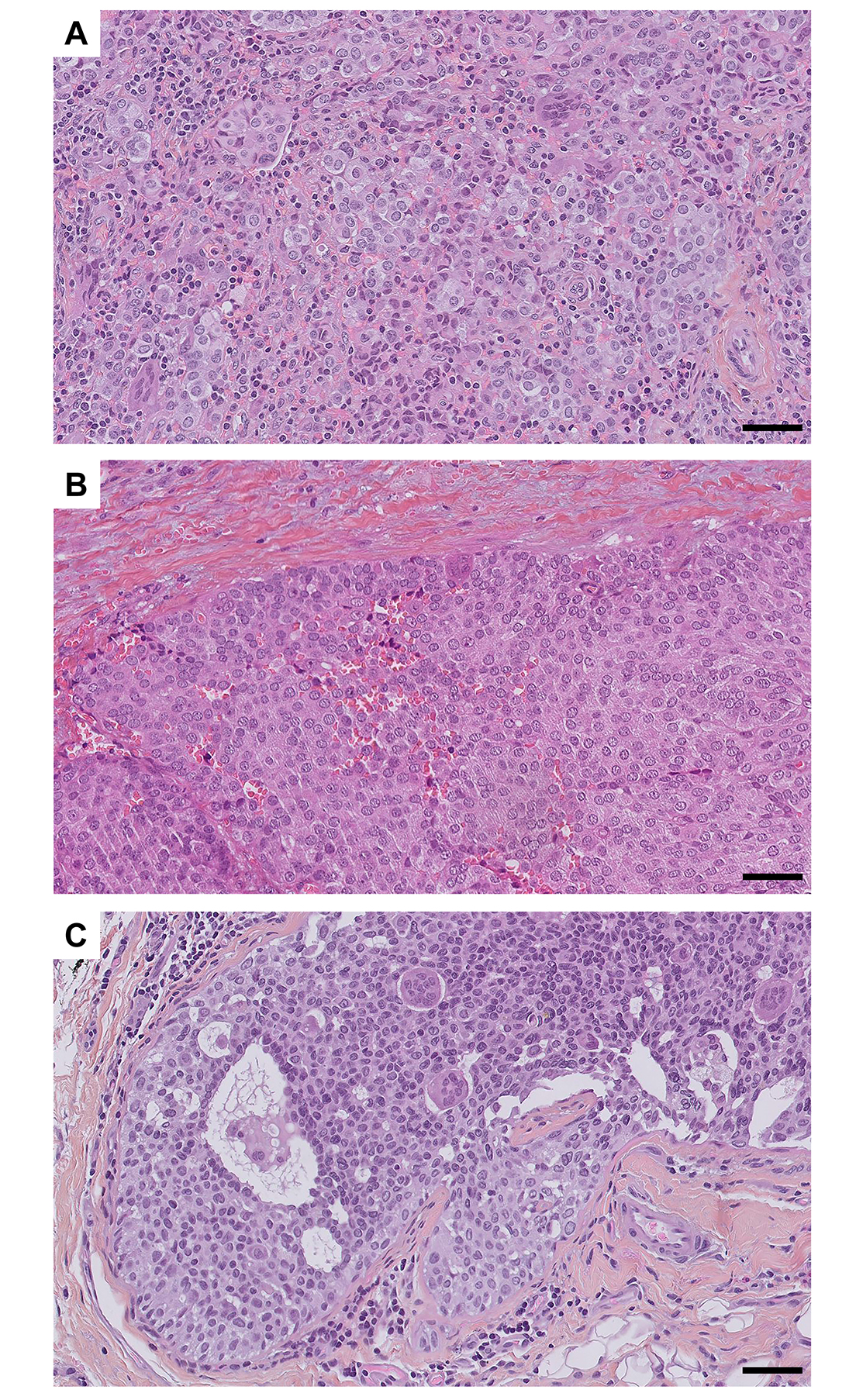
**

**Supplementary Figure S2. Presence of OGC in lymph node metastases and in DCIS.**

**A:** Axillary lymph node metastasis in patient OGC-IC NST 2, showing numerous OGC and erythrocyte extravasation; **B**: Axillary lymph node metastasis in patient OGC-IC NST 5, showing rare OGC and erythrocyte extravasation; **C**: DCIS component in the tumor of patient OGC-IC NST 6, with OGC readily present. Hematoxylin-eosin-saffron stain, scale bars: 50 μm.

**Supplementary Figure S3**

**
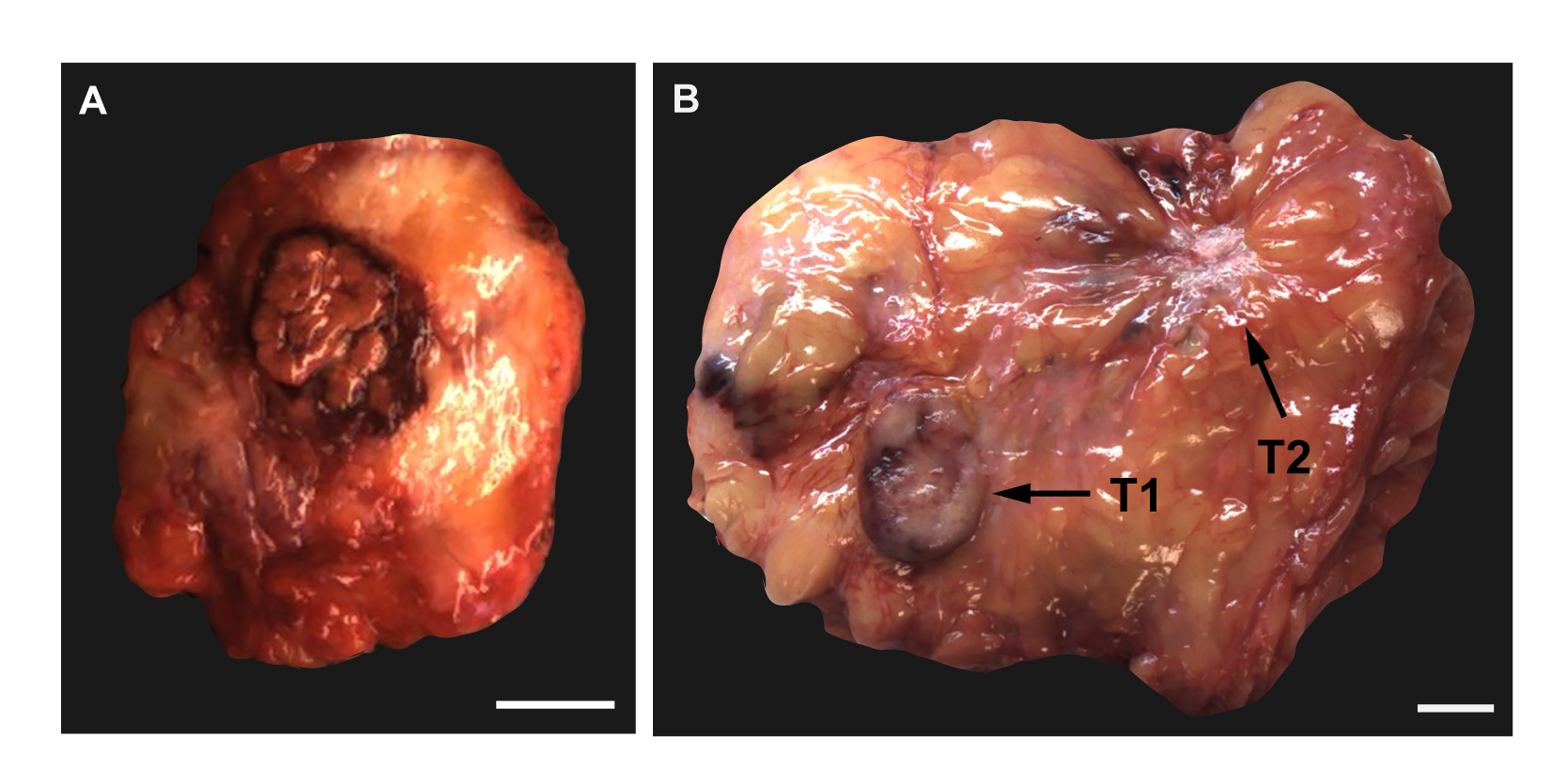
**

**Supplementary Figure S3. Macroscopic appearances of BC with OGC.** **A:** case “OGC-IC NST 13”, showing typical features of BC with OGC: a well-circumscribed, “rusty” tumor with a hemorrhagic rim; **B:** case “OCG-Mixed 2”, showing bifocal disease: IC NST with OGC (tumor T1), presenting as a well-circumscribed tumor with a hemorrhagic rim, and IC NST without OGC (tumor T2), presenting as a stellar, whitish lesion. IC NST: invasive carcinoma of no special type, OGC: osteoclast-like giant cells. Scale bars, 1 cm.

**Supplementary Figure S4**

**
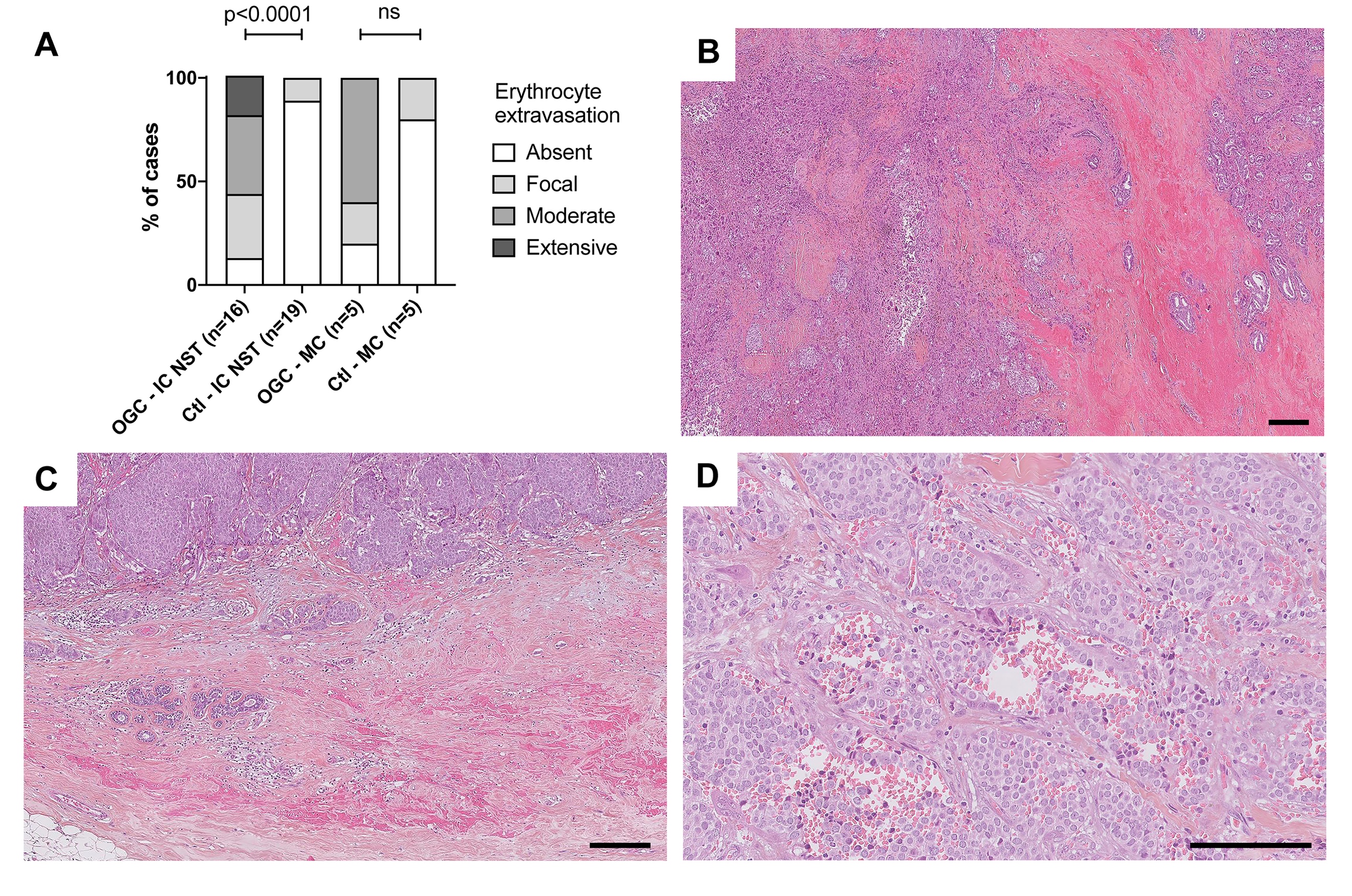
**

**Supplementary figure S4. Erythrocyte extravasation in BC with OGC. A.** Semi-quantitative assessment of extravasation across subgroups. Statistical comparison (Fisher’s exact test) was done between OGC - IC NST and Ctl - IC NST, comparing the proportion of cases with extravasation (any amount) and without extravasation (“absent”). **B-C.** Examples of extravasation in case OGC-CI NTS 5 (**B**), OGC-IC NST 8 (**C**) and OGC-IC NTS 2 (**D**). Scale bars, 100 μm.

**Supplementary Figure S5**

**
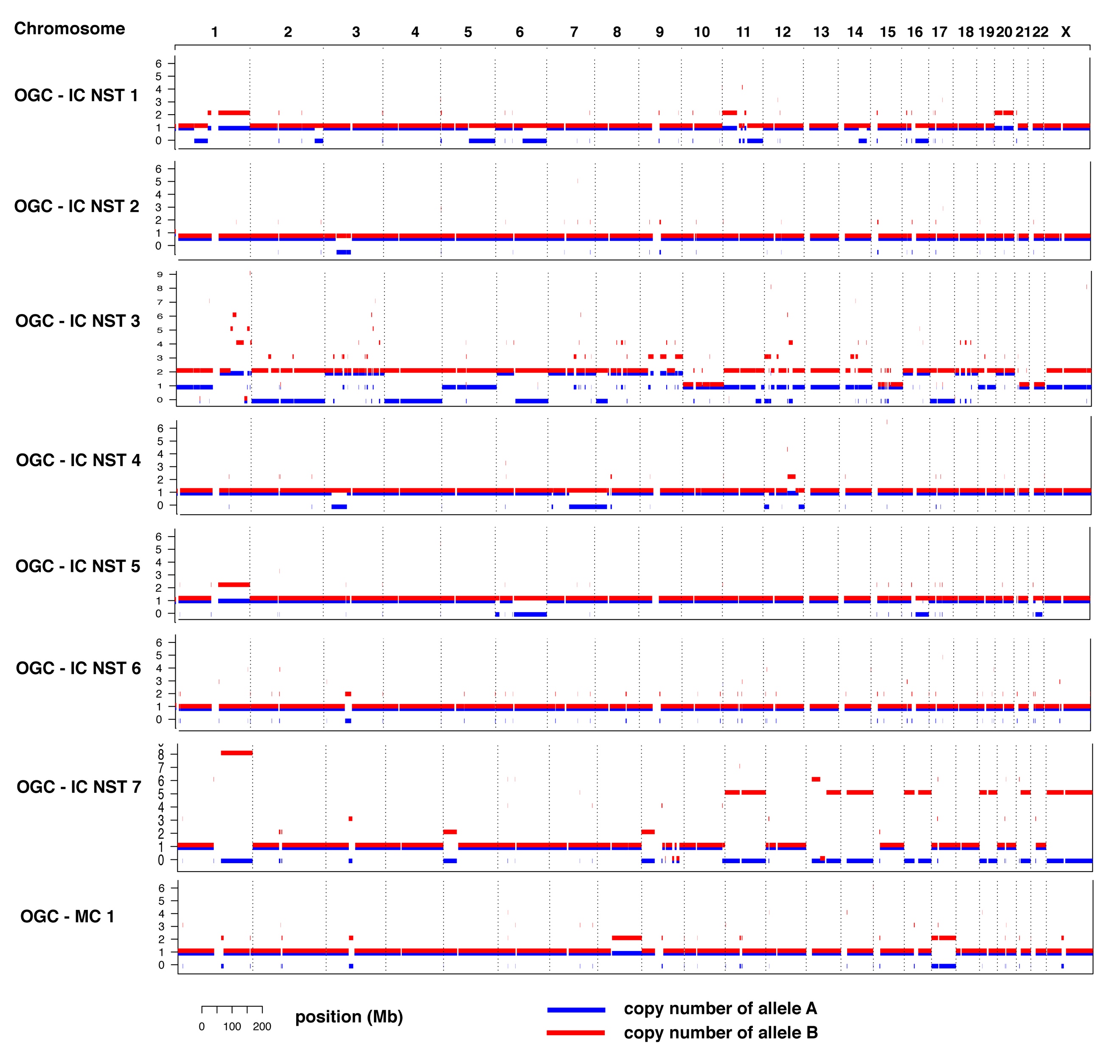
**

**Supplementary figure S5. Whole exome-based copy number alteration (CNA) profiles in BC with OGC.** The CNA profiles were generated using Sequenza (v.2.1.1). OGC-IC NST: invasive carcinoma of no special type with osteoclast-like giant cells; OGC-MC: metaplastic carcinoma with osteoclast-like giant cells; Mb: megabases; the “y” axis shows copy number estimates expressed as absolute values.

**Supplementary Figure S6**

*
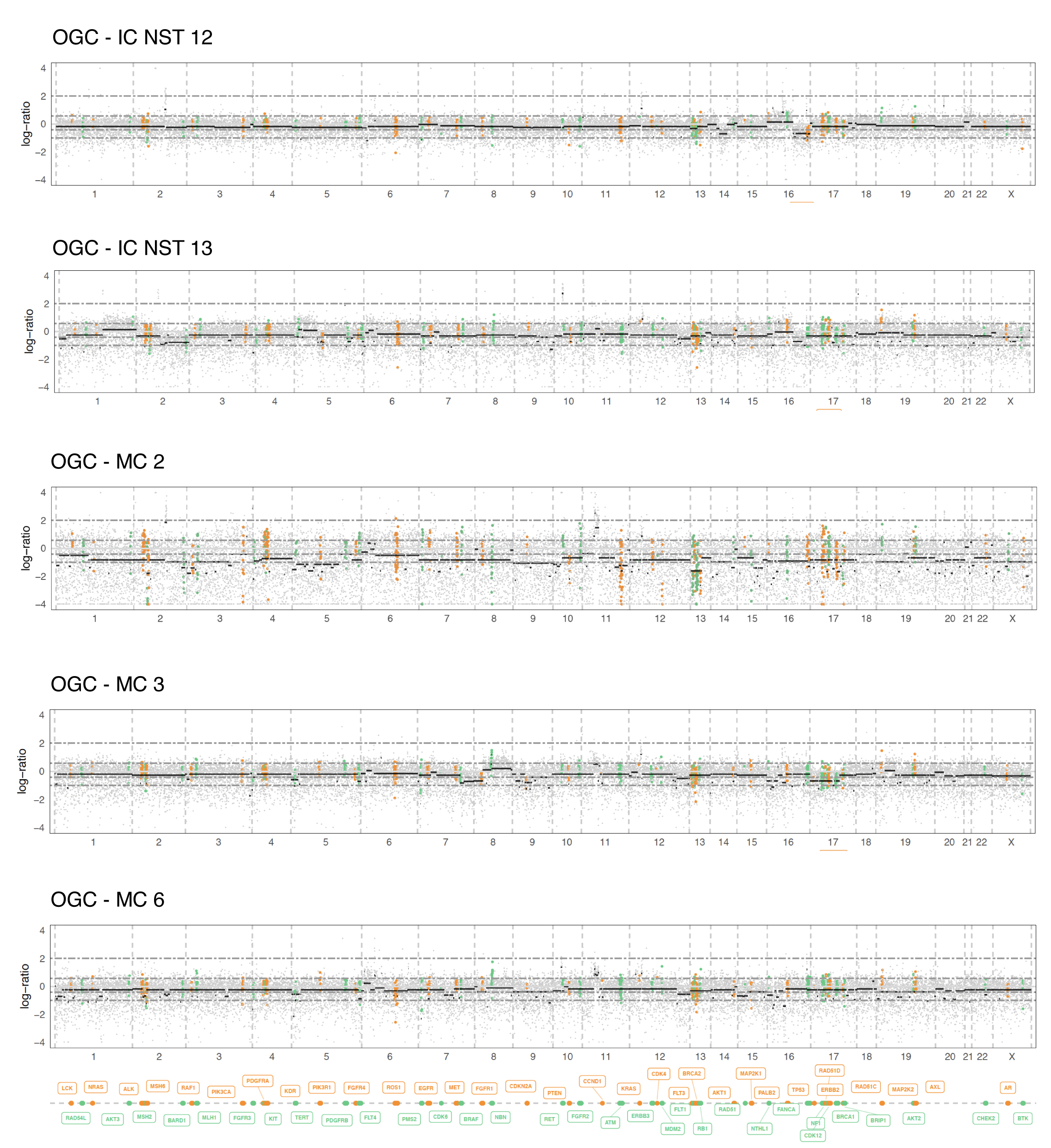
*

**Supplementary figure S6. Targeted DNA sequencing-based copy number alteration (CNA) profiles in BC with OGC.** OGC-IC NST: invasive carcinoma of no special type with osteoclast-like giant cells; OGC-MC: metaplastic carcinoma with osteoclast-like giant cells; the “x” axis represents consecutive chromosomes; the “y” axis shows copy number, expressed as log2 ratio.

**Supplementary Figure S7**

**Supplementary figure S7. Gene Ontology overrepresentation analysis using GO_BP**

**(Biological Process) gene sets.**

**Supplementary Figure S8**

**Supplementary figure S8. Gene Ontology overrepresentation analysis using GO_CC**

**(Cellular Component) gene sets.**

**Supplementary Figure S9**

**Supplementary figure S9. Gene Ontology overrepresentation analysis using GO_MF**

**(Molecular Function) gene sets.**

**Supplementary Figure S10**

**
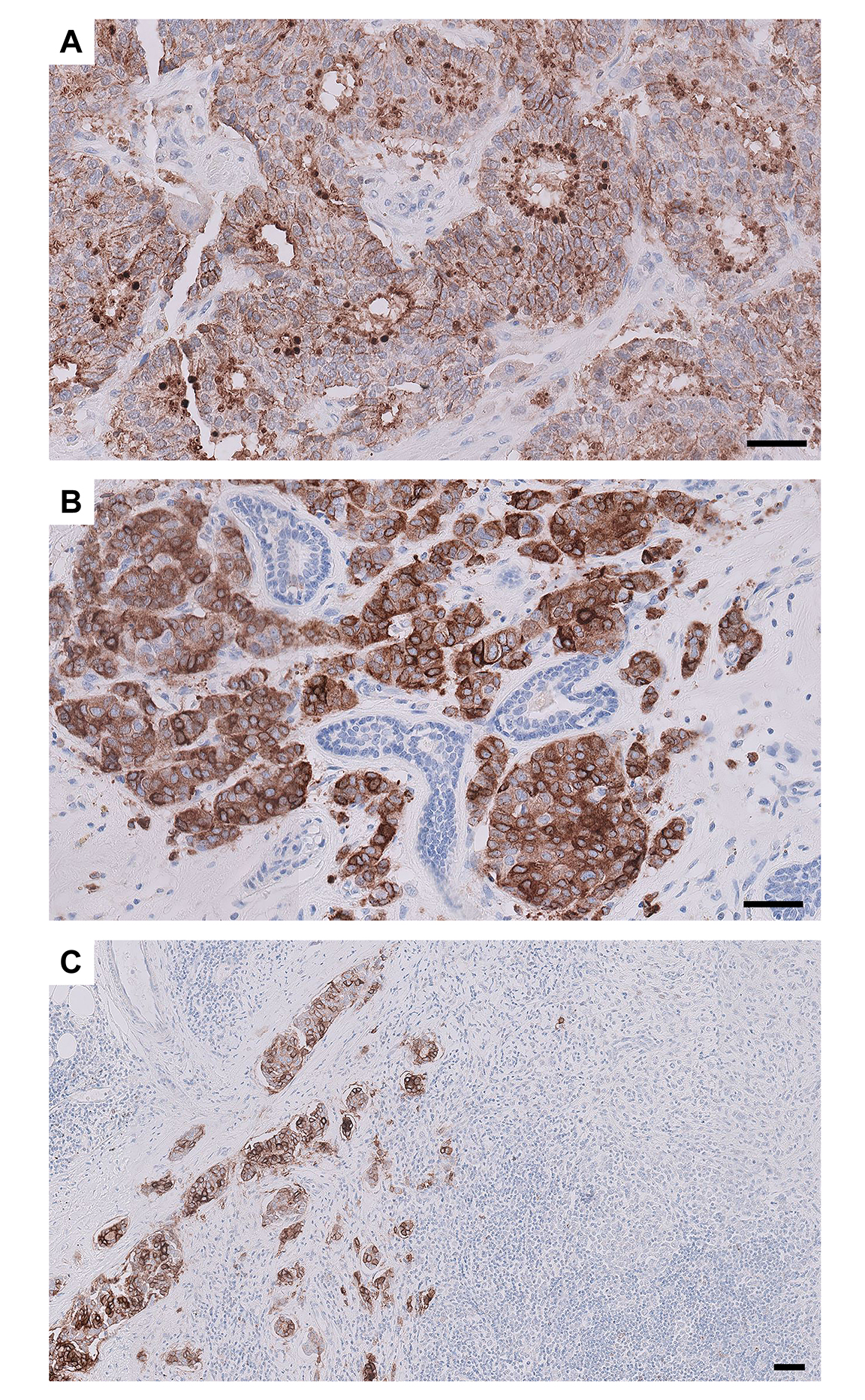
**

**Supplementary figure S10. Additional examples of RANK-L immunostaining.**

**A:** Case OGC - IC TNS 12, showing membranous staining, as well as strongly RANKL-positive vesicles close to the apical membrane; **B:** OGC - IC TNS 8, showing moderate to strong membranous and cytoplasmic staining in tumor cells, while benign epithelial structures were negative in this case; **C**: Axillary lymph node metastasis from patient OGC-Mixed 1, showing areas of RANKL-positive (left) and RANKL-negative (right) metastatic tumor. Scale bars, 50 μm

**Supplementary Figure S11**

*
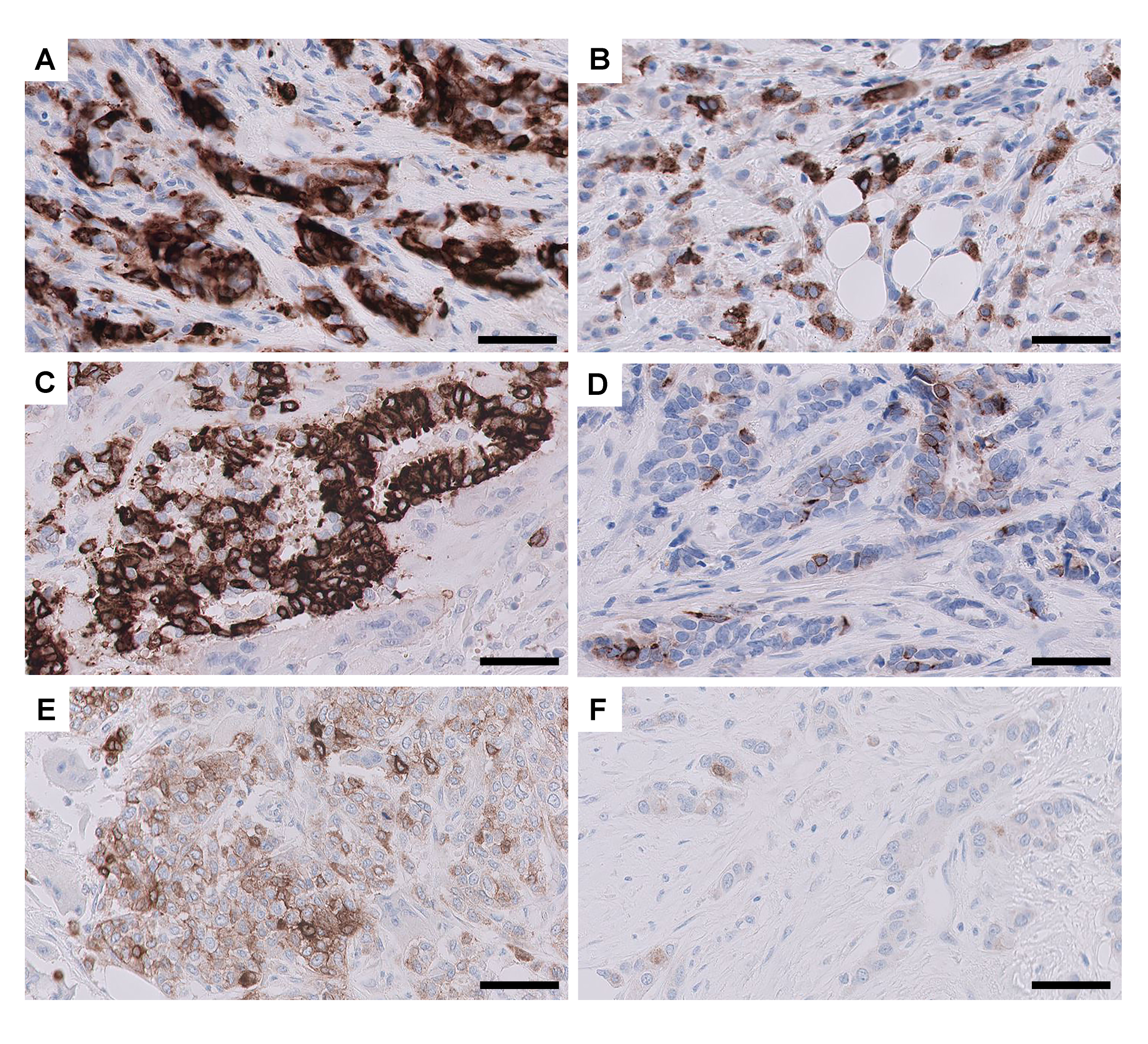
*

**Supplementary figure S11. RANK-L immunostaining in “mixed” cases with and without OGC. A** and **B.** Case OGC-Mixed 3, invasive carcinoma component with (**A**) and without (**B**) OGC; **C** and **D.** Case OGC-Mixed 2, invasive carcinoma component with (**C**) and without (**D**) OGC; **E** and **F.** Case OGC-Mixed 1, invasive carcinoma component with (**E**) and without (**F**) OGC. Scale bars, 50 μm.

**Supplementary Figure S12**


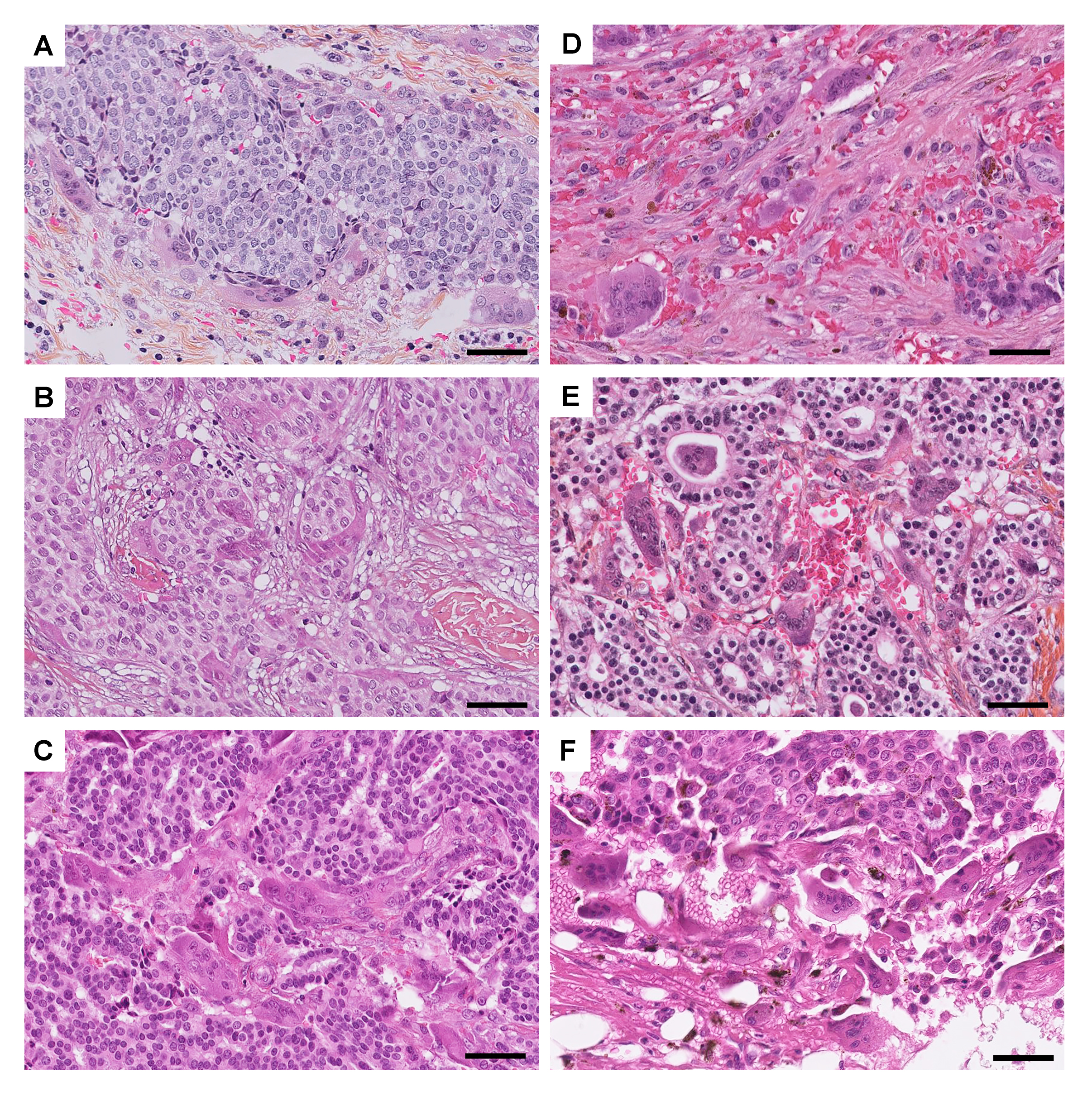


**Supplementary figure S12. A-C:** additional images of OGC “molding” around tumor clusters.

**D-E:** additional images of vascular lacunae around OGC. Cases: OGC-IC TNS 14 (A), OGC-IC TNS 2 (B), OGC-IC TNS 10 (C), OGC-IC TNS 13 (D), OGC-Mixed 4 (E), OGC-IC TNS 11 (F). Hematoxylin-eosin-saffron stain, scale bars: 50 μm.
