# Supplementary material for: Breast carcinomas with osteoclast-like giant cells: a comprehensive clinico-pathological and molecular portrait and evidence of RANK-L expression
Source: Mod Pathol. 2022 Jun 13;35(11):1624–35. doi: 10.1038/s41379-022-01112-9 (PMC9596373; doi:10.1038/s41379-022-01112-9)
Supplement: Supplementary file 3 — Supplementary methods [file 41379_2022_1112_MOESM3_ESM.docx]

**Supplementary Methods**

**Breast carcinomas with osteoclast-like giant cells: a comprehensive clinico-pathological and molecular portrait and evidence of RANK-L expression**

Joanna Cyrta^1,2,@^, Camille Benoist^3^, Julien Masliah-Planchon^4^, Andre F. Vieira^1^, Gaëlle Pierron^4^, Laetitia Fuhrmann^1^, Camille Richardot^1^, Martial Caly^1^, Renaud Leclere^1,5^, Odette Mariani^1^, Elisabeth Da Maia^6^, Frédérique Larousserie^7^, Jean Guillaume Féron^8^, Matthieu Carton^9^, Victor Renault^3^, François-Clément Bidard^10^, Anne Vincent-Salomon^1^

^1^Department of Pathology, Institut Curie, PSL Research University, Paris, France

^2^Université de Paris, Paris, France

^3^Clinical Bioinformatics Unit, Institut Curie, PSL Research University, Paris, France

^4^Somatic Genetics Unit, Institut Curie, Paris, France

^5^Platform of Experimental Pathology PATHEX, Institut Curie, Paris, France.

^6^Department of Pathology, Hôpital de la Pitié-Salpêtrière, Paris, France

^7^Department of Pathology, Hôpital Cochin, AP-HP, Université Paris Cité, Paris, France

Hôpital Cochin, Paris, France

^8^Department of Surgery, Institut Curie, Paris, France

^9^Department of Biometry, DRCI, Institut Curie, PSL Research University, Paris, France

^10^Department of Medical Oncology, Institut Curie, UVSQ/Paris-Saclay University, St Cloud, France

^@^ Correspondence to: joanna.cyrta@curie.fr and anne.salomon@curie.fr

**Perls Prussian blue stain**

Perls Prussian blue stain was performed by incubating deparaffinized and rehydrated tissue sections with 2% potassium ferrocyanide and 2% and 2% hydrochloric acid during 30 min. After rinsing in distilled water, slides were counter-colored with nuclear red.

**Gene Set Enrichment Analysis (GSEA)**

Gene Set Enrichment Analysis (GSEA) was performed using the GSEA application for Mac v4.2.3 ^1,2^. Genes were first pre-ranked according to values of -log(adjusted p-value)*sign(log2FoldChange), whereby “sign” represents “-1” for genes with negative log2FoldChange and “1” for genes with positive log2FoldChange values, respectively. This resulted in a pre-ranked list containing a total of 14630 features (genes). The number of permutations was set at 1000 and the platform was set as Human_ENSEMBL_Gene_ID_MSigDB.v7.5.1. GSEA was performed for the following gene sets from the Molecular Signatures Database (MSigDB) ^3^: C5_BP (Biological Processes), C5_CC (Cellular Component) and C5_MF (Molecular Function); C2 (Curated genesets); C6 (Oncogenic Signatures); H (Hallmark). Results with NOM p-value<0.05 and FDR q-value<0.25 were considered significant.

**Gene Ontology**

Gene Ontology was performed using the enrichGO() function with ont="BP" (for biological processes), "MF" (for molecular functions), "CC" (for cellular components) from R package *clusterProfiler* ^4^*.*A statistical test is applied to verify if the genes of interest are more often associated to certain biological functions (GO terms) than what would be expected in a random set of genes from the genome.

**Molecular subtype prediction**

Subtype prediction was performed using the subtype.cluster.predict() fuction (sbt.model="pam50") from R package *genefu* version 2.16.0 ^5^. This is based on the intrinsic subtyping classifier that measures expression of 50 genes selected as characteristic of the five breast cancer intrinsic subtypes ^6^. To classify intrinsic subtypes from the gene expression data, centroid-based algorithms are applied to the calibrated log-expression ratio for the 50 genes. This process generates, for each sample, five continuous-scale normalized subtype scores representing degree of correlation of gene expression with that of archetypal Luminal A, Luminal B, HER2-enriched, Basal-like, and Normal-like breast tumors. Tumors were classified as the subtype with the highest normalized subtype score.

**Supplementary references**

1 Mootha, V. K. *et al.* PGC-1alpha-responsive genes involved in oxidative phosphorylation are coordinately downregulated in human diabetes. *Nat Genet* **34**, 267-273 (2003).

2 Subramanian, A. *et al.* Gene set enrichment analysis: a knowledge-based approach for interpreting genome-wide expression profiles. *Proc Natl Acad Sci U S A* **102**, 15545-15550 (2005).

3 Liberzon, A. *et al.* The Molecular Signatures Database (MSigDB) hallmark gene set collection. *Cell Syst* **1**, 417-425 (2015).

4 Yu, G., Wang, L. G., Han, Y. & He, Q. Y. clusterProfiler: an R package for comparing biological themes among gene clusters. *OMICS* **16**, 284-287 (2012).

5 Gendoo, D. M. *et al.* Genefu: an R/Bioconductor package for computation of gene expression-based signatures in breast cancer. *Bioinformatics* **32**, 1097-1099 (2016).

6 Parker, J. S. *et al.* Supervised risk predictor of breast cancer based on intrinsic subtypes. *J Clin Oncol* **27**, 1160-1167 (2009).
